# Hybrid Particle Swarm Optimization for Multi-Sensor Data Fusion

**DOI:** 10.3390/s18092792

**Published:** 2018-08-24

**Authors:** Hyunseok Kim, Dongjun Suh

**Affiliations:** 1 IoT Research Division, Electronics and Telecommunications Research Institute (ETRI), Daejeon 34129, Korea; hertzkim@etri.re.kr; 2 School of Convergence & Fusion System Engineering, Kyungpook National University, Sangju 37224, Korea

**Keywords:** multi-sensor system, multi-sensor information fusion, particle swarm optimization, sensor data fusion algorithm, distributed intelligence system

## Abstract

A hybrid particle swarm optimization (PSO), able to overcome the large-scale nonlinearity or heavily correlation in the data fusion model of multiple sensing information, is proposed in this paper. In recent smart convergence technology, multiple similar and/or dissimilar sensors are widely used to support precisely sensing information from different perspectives, and these are integrated with data fusion algorithms to get synergistic effects. However, the construction of the data fusion model is not trivial because of difficulties to meet under the restricted conditions of a multi-sensor system such as its limited options for deploying sensors and nonlinear characteristics, or correlation errors of multiple sensors. This paper presents a hybrid PSO to facilitate the construction of robust data fusion model based on neural network while ensuring the balance between exploration and exploitation. The performance of the proposed model was evaluated by benchmarks composed of representative datasets. The well-optimized data fusion model is expected to provide an enhancement in the synergistic accuracy.

## 1. Introduction

**Figure 1:**
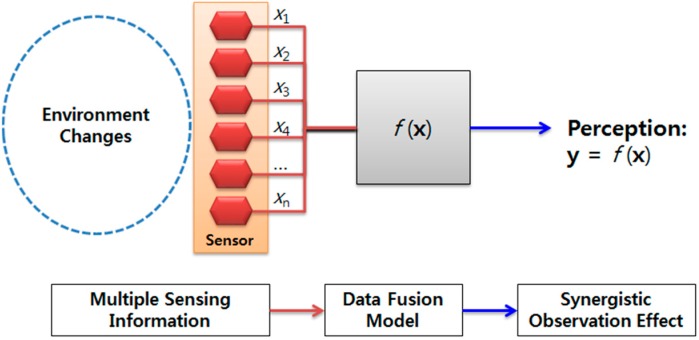
A multi-sensor system.

A moment of evolution is now emerging toward a new paradigm known as smart convergence, which is bringing together both heterogeneous and information communication technologies. These emerging phenomena have prompted researchers to explore new possibilities for sophisticated smart devices [1] to be embedded in various real objects and to cope with various environmental changes. Recently, multiple similar and/or dissimilar sensors, as shown in Figure 1, have been widely used to provide precise sensing information from different viewpoints [2,3] and to realize the Internet of Things in a cyber-physical system [4]. Given that the accuracy of a sensor system is dictated by the degree to which repeated measurements under unchanging conditions are able to produce the same results, a multi-sensor system has typically been thought of as a way to guarantee the accuracy of a measurement system [5]. Hence, multi-sensor systems are an emerging research topic that is becoming increasingly important in various environmental perception activities. Nevertheless, challenging problems of multisensory data fusion algorithms are still far from accomplished [6]. Evolutionary computation methods [7] recently seem to be making a comeback in order to solve real-world problems concerning typically not iid (independent and identically distributed) data or sparse labeled data, and these methods are expected to help such a fusion model enhanced.

Because a single sensor usually only recognizes a limited set of partial information about the environment, multiple similar and/or dissimilar sensors are needed to provide accurate sensing information from a variety of perspective in an integrated manner [8]. Such multiple-sensing information is combined depending on data fusion algorithms to achieve synergistic effects. However, the construction of a data fusion model is not trivial because of difficulties in meeting the restricted conditions of a multi-sensor system such as its limited options for deploying sensors and nonlinear characteristics or correlation errors of multiple sensors. Such a nonlinearity optimization problem in a data fusion model can be solved by a neural network algorithm with an effective back propagation method ensuring the best performance of the network [9]. In recent multi-sensor fusion research [10,11], neural networks (including deep neural networks) play a major role in feature classification and decision making. However, difficulties for the efficient and high accurate multisensory fusion model remain. 

As is well known, maintaining the balance between the exploration of new possibilities and the exploitation of old certainties [12,13] has been considered a priority in designing an optimization scheme. As one of latest evolutionary approaches, particle swarm optimization (PSO) uses randomly placed particles on the search-space to explore new possibilities toward a global best position as a new solution to solve real-world problems [14,15,16]. PSO can be utilized in training a neural network by a population based stochastic back-propagation technique [17]. The randomly placed particles are more likely to find the global minimum than neural networks using a single particle. Venu et al. [18] showed that the parameters of feed-forward neural network converge faster using PSO than any other algorithm based on back-propagation methods, e.g., stochastic gradient descent, scaled gradient descent, or Levenberg-Marquardt (LM) [19]. They adopted PSO as a training algorithm involving adjusting the parameters (i.e., weights and biases) to optimize performance of the neural network. Kim et al. [20] suggested the PSO proportional-integral-derivative (PSOpid) which is one of the enhanced PSO algorithms through the stabilization of particle movement. Although a particle can be used as one of the solutions to regulate the parameters of neural network, it is necessary to constrain the range of search space for quicker convergence and higher fitness in PSO. 

This paper proposes a hybrid PSO capable of overcoming large-scale nonlinearity or heavy correlation in the sensing data fusion and facilitates the construction of a robust data fusion model while maintaining the balance between the exploration of new possibilities and the exploitation of old certainties. The proposed algorithm was evaluated by benchmarks composed of representative datasets and the well-optimized data fusion model is expected to provide an enhancement in synergistic accuracy. In this paper, the neural network is used as a basic model and different backpropagation methods such as PSO, LM, and PSOpid are considered, thus the expression of neural network is simply omitted.

Section 2 discusses the problem of data fusion model and previous methods such as ordinary LM and PSO. We then propose a hybrid PSO, LM, and PSOpid in Section 3, and Section 4 describes how evaluation is performed for the proposed approach with the different weighing participants in diverse user-scenarios and with the different exemplary datasets from MATLAB. Section 5 concludes the research and describe possible future works.

## 2. Data Fusion Model Using Neural Network and PSO

Compared to a single sensor system, multiple sensor systems have the advantage of broadening the sensing range and improving the perception of environmental conditions [8]. In addition, a multi-sensor system allows information from a set of homogeneous sensors to be combined using a data fusion model. Thus, a multi-sensor system represents a proven method for enhancing the accuracy of a measurement system.

**Figure 2:**
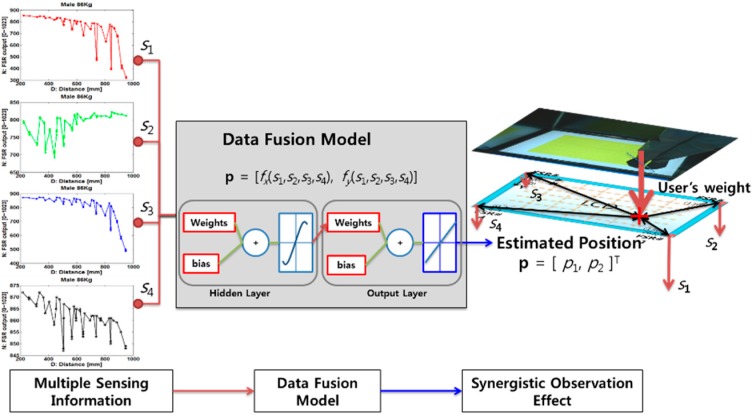
Example of user localization using a multi-sensor system.

Figure 2 shows the case of using multiple Force Sensitive Resistor (FSR) sensors on a smart floor block [16]. FSRs is one of best solutions to meet multi-dimensional requirements such as maintaining visibility; holding weights of standing, moving, or jumping user; avoiding occlusion in sensing area; and reducing production cost. In this paper, an FSR is composed of two substrate layers with a conductive core, and, when pressure is applied to the FSR, the substrate moves, compressing the conductive core to detect the weight on the smart floor. The pressure changes are measured by the analog output from the FSR resistive divider. The weight is dispersed to the corner based on the approximate inverse power law which is the relation between distance and force of the FSR [21]. 

In [16], a neural network adopting three layers is used, and we can clarify our notation and describe it as follows. Each four-dimensional vector is applied to the input layer as $s={\left[\begin{array}{cc} \begin{array}{cc} s_{1} & s_{2} \end{array} & \begin{array}{cc} s_{3} & s_{4} \end{array} \end{array}\right]}^{T}$, where $\left\{s\in \mathbb{R}:0\leq s\leq 2^{10}\right\}$. An output produced by a non-linear activation function at each hidden unit, g(net), that is, a hyperbolic tangent sigmoid as,

(1) $$g\left(net\right)=\frac{e^{net}-e^{-net}}{e^{net}+e^{-net}}$$

where the net is the inner product of inputs with weights at each neural network unit.

Each output of the neural network as $p={\left[p_{1}\;p_{2}\right]}^{T}$ calculates the activation function based on the hidden units described in Equation (2):

(2) $$p_{k}\left(s\right)\equiv \sum_{j=1}^{n_{H}}\theta_{kj}g\left(\sum_{i=1}^{d}\theta_{ji}s_{i}+\theta_{j0}\right)+\theta_{k0}$$

where the subscript *i* indexes units in the input layer and *j* indexes units in the hidden layer; $\theta_{ji}$ denotes the input-to-hidden layer parameters at the hidden unit *j*; and the dimension of input vector is *d*. In addition, the subscript *k* indexes units in the output layer, $n_{H}$ indicates the number of hidden units and $\theta_{kj}$ denotes the hidden-to-output layer weights. $\theta_{j0}$ and $\theta_{k0}$ are mathematically treated as the bias of the layer. We refer to its output, $p={\left[p_{1}\;p_{2}\right]}^{T}$, as the user’s estimated 2D position (*x*, *y*) on the screen, respectively. The authors in [16] regarded the cost function as the sum of the output units of the squared difference between the target $t_{k}$ and the actual output $a_{k}$.

(3) $$J\left(\theta \right)\equiv \frac{1}{2m}\sum_{i=1}^{m}\sum_{k=1}^{c}{\left(t_{k}-a_{k}\right)}^{2}=\frac{1}{2m}\sum_{i=1}^{m}{∥t-a∥}^{2}$$

where **t** and **a** are the target and actual output vectors of length *c* (e.g., *c* is 2 in this experiment), the batch size is *m* and **θ** represents all weights and biases.

FSRs show nonlinear issues or problems of correlation errors. It can be caused by instability of the glass or metal frame, response time of the sensor, errors in manufacturing, assembly processes, the sensor placement, and human errors. Therefore, Kim et al. [16] suggested a PSO-based neural network model, namely PSO, which can be used as alternatives method to the gradient-descent algorithm by randomly spreading multiple particles capable of finding each optimum and being converged toward the global optima. However, if a system needs many parameters that should be adjusted for many epochs and other potential parameters, then the system needs to be improved.

## 3. A Hybrid PSO Model for Multi-Sensor Data Fusion

Since March [12] proposed the balancing between exploration and exploitation in learning, it has been extensively researched and widely applied to various domains [13]. In this paper, the PSO first explores possibilities as a global search, the LM then exploits certainties as a local search, and the PSOpid suggested in [20] lastly explores a new possibility within the range-optimized search space. The PSOpid is one of the enhanced PSO algorithms and each particle finds the global optimum securely while preventing a particle from becoming unstable or exploding. In addition, the algorithm can converge more quickly and get high fitness values compare to other algorithms in the range-optimized search space.

### 3.1. Improved Exploitation of Neural Network Using Ordinary PSO 

The idea of PSO allows particles randomly placed on the search-space to explore new possibilities to the best global position. PSO is used to train a neural network with back propagation method considering a population based stochastic optimization technique [17]. On the other hand, the randomly placed particles will have a high probability of discovering the global minimum in comparison with the ordinary LM using a single particle. In this method, PSO can be alternatively used as the training algorithm at each iteration *n*, including parameter tuning to optimize the performance of the neural network. In addition, a particle is used to determine the value of parameters in the neural network. The magnitude of vectors $\theta_{i}$ and $v_{i}$ are equivalent to the dimension of the weights and biases. The velocity and position of the *i*-th particle after the *n*-th iteration are shown in Equations (4) and (5).

(4) $$v_{i}\left[n\right]=K\left\{\begin{array}{c} \omega \cdot v_{i}\left[n-1\right]\\ +c_{1}\cdot rand_{1}\cdot (p_{i_{\mathrm{best}}}\left[n-1\right]-\theta_{i}\left[n-1\right]\\ +c_{1}\cdot rand_{2}\cdot (g_{best}\left[n-1\right]-\theta_{i}\left[n-1\right] \end{array}\right\}$$

(5) $$\theta_{i}\left[n\right]=\theta_{i}\left[n-1\right]+v_{i}\left[n\right]$$

The previous best position is selected using Equation (6), and the global best position is decided by Equation (7).

(6) $$p_{i_{\mathrm{best}}}\left[n\right]∶=\{\begin{array}{c} \theta_{i}\;\mathrm{if}\;J\left(\theta_{i}\left[n\right]\right)\leq J\left(\theta_{i}\left[n-1\right]\right)\\ p_{i_{\mathrm{best}}}\left[n-1\right]\;\mathrm{otherwise} \end{array}$$

(7) $$g_{\mathrm{best}}\left[n\right]=\left\{p_{j_{\mathrm{best}}}\left[n\right]|\forall i:J\left(p_{j_{\mathrm{best}}}\left[n\right]\right)\leq J\left(p_{i_{\mathrm{best}}}\left[n\right]\right)\right\}$$

where the number of particles is *Z_k_* (≈ 10 + 2 × (the dimension of the weights and biases)^0.5^) and the inertia weight is *ω*, which dynamically adjusts the velocity. Moreover, the cognitive component *c*_1_ and the social component *c*_2_ change the particles’ velocity toward the previous best and global best position, respectively. The PSO uses random numbers determined from a uniform distribution *rand*_1_ and *rand*_2_ to avoid unfortunate states in which all particles quickly settle into an unchanging direction. Consequently, the parameters are updated in accordance with the found global best position.

This paper proposes a hybrid LM and PSO scheme, namely LM-PSO, based on the concept of two-phase evolutionary programming [22]: First, PSO is used to explore a new possibility for overcoming multiple minima, and second, the initial value of the weights and biases of LM-based neural network, namely LM, is set—in other words, the neural network is trained—to the optimal configuration derived in the PSO phase. Consequently, the proposed LM-PSO can derive more accurately a globally optimal solution compared to LM. 

**Figure 3:**
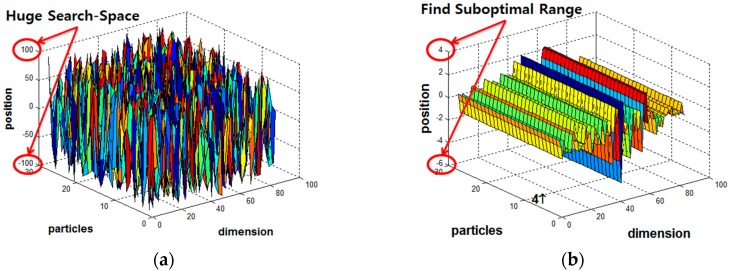
PSO alone: (**a**) Initial position of the particles within a hypercube using a uniform random distribution; and (**b**) converged position of the particles.

In the first stage, normally a PSO has the initial parameters shown in Table 1 for a multiple FSR system. As illustrated in Figure 3, PSO finds suboptimal range of parameters which can reduce the configuration space for LM’s parameters.

In the second stage, the proposed LM-PSO is trained with the optimal initial weight and bias values derived during the PSO stage. Although PSO is implausible on its own as a solution for the convergence rate and solution accuracy as illustrated in Figure 3, the proposed hybrid LM-PSO shows an enhanced performance of both accuracy and convergence rate as compared to conventional LM. However, hope remains for new possibilities for deriving an ultimate global optimum.

### 3.2. Exploration Toward Ultimate Goals for the Use of Enhanced PSO 

Ordinary PSO alone has difficulty negotiating the tradeoff between global and local search because particles are initially deployed following a uniform random distribution in a hypercube big enough to contain a prospective global optimum. When the particle position range is not limited within the minimum and maximum positions or is too broad, the swarm can become unstable or explode, resulting in a slow convergence. Therefore, a novel approach is necessary for each particle to discover the global optimal solution securely, preventing the particles from becoming unstable or exploding.

The proportional-integral-derivative (PID) is widely applied in a feedback control loop technology [23]. The major advantages of the PID are easy to be implemented as well as only three parameters, i.e., proportional, integral and derivative terms, are required to be adjusted. The proportional gain is subject to the current error, the integral gain varies in proportion to both the magnitude and the duration error, and the derivative gain represents a prediction of future errors [24]. In [20], Kim et al. proposed a new approach using PSOpid as described in Equation (8) to change the particle’s position of PSO with less oscillation.

(8) $$\theta_{i}\left[n\right]=\theta_{i}\left[n-1\right]+\left\{\begin{array}{c} K_{P}\cdot v_{i}\left[n\right]\\ +K_{I}\cdot \sum_{k=0}^{n}v_{i}\left[k\right]\\ +K_{D}\cdot \left(v_{i}\left[n\right]-v_{i}\left[n-1\right]\right) \end{array}\right\}$$

where *K_P_* is the proportional term, *K_I_* is integral term, and *K_D_* is the derivative term, and they are selected through trial and error operations, as shown in Table 2.

Adjustment of the velocity is calculated from the acceleration with respect to the distance error produced by both present and its previous best position compared to the global best solution. Furthermore, two random, uniformly distributed variables are utilized in preserving the diversity. The position of each particle is updated according to the multiplying the velocity with the three PID terms, which are described in Table 2. In addition, it is necessary to restrain the dynamic range of each particle position to prevent a swarm from exploding or unstable conditions.

**Figure 4:**
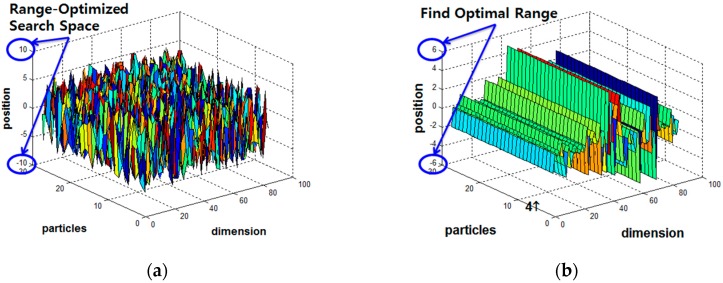
A hybridization of PSOpid, LM and PSO, namely PSOpid-LM-PSO: (**a**) initial position of particles within a shrunk hypercube; and (**b**) convergence position of the particles.

The position of each particle is initialized to the range between maximum and minimum derived from the output of the precedent LM-PSO technique. The initial global best position is optimally configured in accordance with the LM-PSO method. Consequently, particles randomly distributed can better guarantee an accurate global optimal solution than they can through either method alone, as shown in Figure 4.

The stabilized PSOpid, in which each particle is evaluated by using a fitness function and update, is located in an enhanced location to discover the best optimal solution without local minimum. The convergence rate of the proposed PSOpid is faster and it has much higher performance than previous methods. This technique enables efficient implementation because of its small number of parameters, which both shortens the training time and reduces overfitting compared to ordinary PSO. 

### 3.3. Three-Phase Hybrid PSO Method Balancing between Exploration and Exploitation

**Figure 5:**
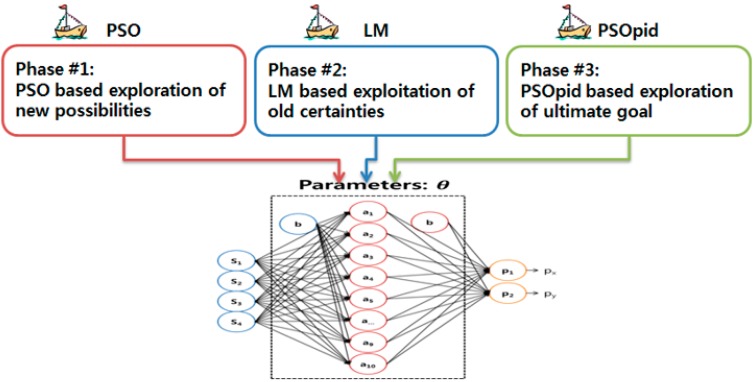
Three-phase hybrid optimization method.

The proposed PSOpid-LM-PSO, as shown in Figure 5, assigns each particle a position calculated based on the output of the LM-PSO phase to initialize an optimal configuration of a global best position. The swarm intelligence technology, in which each individual evaluates, compares, and imitates one another, is a better way to find the best optimum with no local minima. Importantly, the dynamic range of each particle position is limited to prevent a swarm from becoming unstable or exploding. In the three-phase hybrid optimization method, PSO-based exploration of new possibilities is firstly executed for the configuration space of parameters in the multi-sensor data fusion model. Secondly, ordinary LM configured with the sub-optimized range can focus on the exploitation of old certainties. Finally, PSOpid can explore the ultimate goal with more accuracy and faster convergence rate. As a result of the hybrid method, the time needed for convergence is also shorter than that of an ordinary method. Thus, the number of iterations required for convergence counts in comparing speed of convergence.

**Figure 6:**
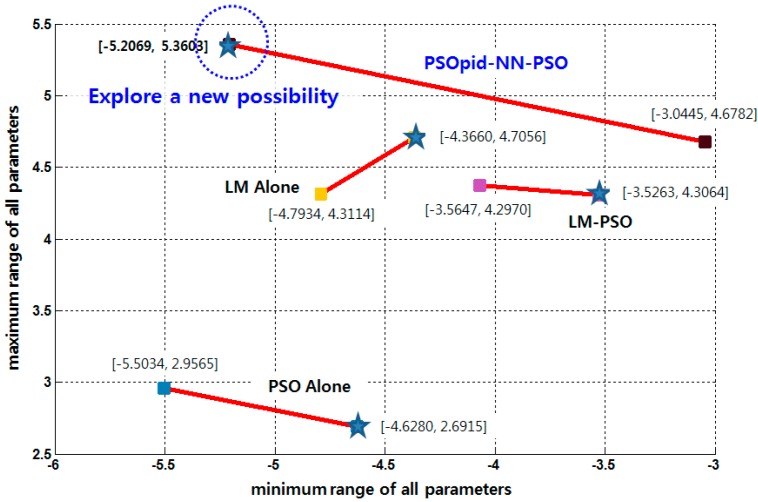
Exploration of a new possibility. Each (x,y) coordinate indicates the minimum and maximum of all parameters such as weights and biases, and the outcomes are from each independent trial.

In the case of PSO and PSOpid alone, the range of each particle position become unstable or explodes; therefore, the hybrid of three algorithms can be used to not only exploit old certainty optimum but also to explore new possibilities. Through all evaluative processes, this paper shows the enhanced performance by a hybrid scheme of ordinary PSO, LM, and PSOpid, namely PSOpid-LM-PSO. The results showed that the proposed PSOpid-LM-PSO provides faster convergence and better fitness than other algorithms within the range-optimized search space, as shown in Figure 6. Each coordinate in Figure 6 indicates the minimum and maximum of the range for parameters in multi-sensor data fusion model. This well-optimized data fusion model for the sensing information of a set of homogeneous FSRs is expected to provide an enhancement in synergistic accuracy.

## 4. Performance Analysis

**Figure 7:**
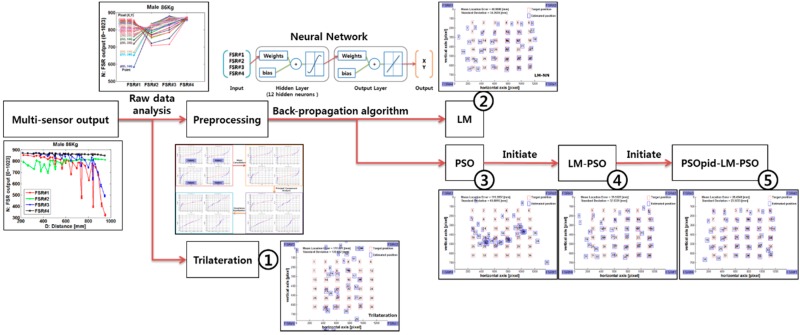
Performance analysis.

We first evaluated the experimental dataset of the touch floor system in [16] and selected men and women from 58 kg to 90 kg as participants. While a user is standing on the floor, the user weight is distributed to four corners according to the inverse power-law in the distance vs. force relation, and the force on each FSRs is sampled through a resistive divider with noise filters. Therefore, it is reasonable to perform experiments with various weighing participants. In this experiment, we evaluated the mean distance error between prediction user location and test set location. As shown in Figure 7, five different algorithms, geometrical trilateration [25], ordinary LM-based neural network (LM), PSO-based neural network (PSO), two phases method (LM-PSO), and the proposed three-phase hybrid method (PSOpid-LM-PSO), are evaluated to investigate whether the suggested method is sufficiently robust to identify the position of differently weighing participants in realistic indoor conditions. In the evaluation, each algorithm was trained with same sized learning data consisting of 100 steps per each participant with 4 × 185 matrix. In addition, these algorithms used the same normalized input data via preprocessing with mean cancellation, principle component analysis and covariance equalization. 

Table 3 presents the results from a comparison test consisting of 100 independent trials. In each trial, an inner random number generator was initialized with a nonnegative integer based on the current clock time, and, thus, the randomized functions can produce a predictable sequence of numbers. As described in Table 3, the estimation error of the proposed PSOpid-LM-PSO is reduced by approximately 88.77% in comparison with the trilateration method. (e.g., in 58 kg case, error reduction is calculated as follows: (1.0 − 23.18 mm/546.07 mm) × 100 = 95.76%).

**Figure 8:**
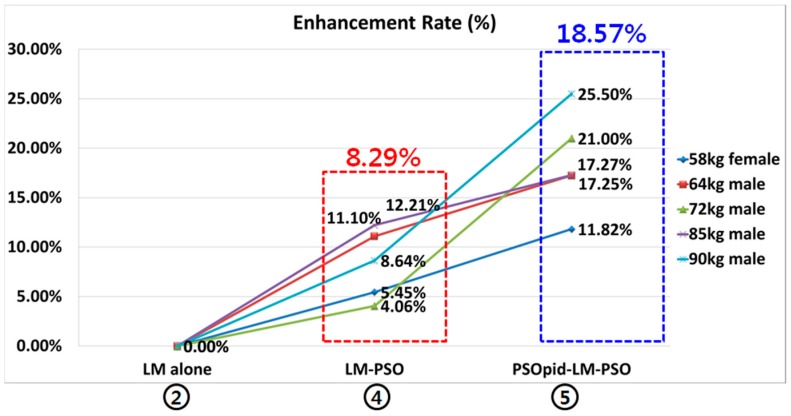
Enhancement graph of each algorithm as compared to the classic LM-based backpropagation algorithm.

Figure 8 shows the rate of enhancement under the PSOpid-LM-PSO in comparison with the classic LM. The PSOpid-LM-PSO method decreased the mean location error by about 18.57% compared to the LM alone case and the overall performance was enhanced [16].

**Figure 9:**
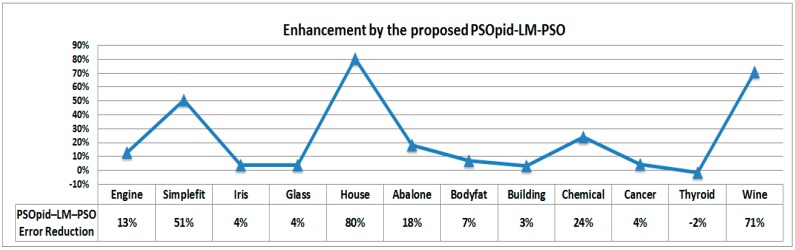
Performance test using a sample dataset.

In contrast, a performance analysis was performed using different exemplary datasets from MATLAB [26], which are widely used to evaluate the performance of machine learning, to confirm that the proposed method is robust enough to improve stability, robustness and convergence speed in terms of neural network training. In this study, we used the three values of the PID (*K_P_*, *K_I_*, and *K_D_*) which are fixed to 0.5, 0.4, and 0.3, respectively to verify the reliability of performance evaluation of the proposed methodology in different exemplar datasets. Other parameters were used in the same manner with the generic PSO and the PSOpid approach. Figure 9 shows the enhancement of hybridizations among PSOpid-LM-PSO and the other algorithms. The results are summarized with the best, median, and worst results (out of 100 independent runs) reported. In the case of PSO and PSOpid, the range of each particle position becomes unstable or explodes; therefore, neither algorithm alone can be used to exploit the old certainty optimum as well as explore new possibilities. Through all evaluative processes, the proposed PSOpid-LM-PSO showed enhanced performance by using a hybrid scheme of ordinary PSO, LM, and PSOpid.

## 5. Conclusions

This study proposed a hybridization of enhanced particle swarm optimization (PSO) and a classic neural network to build a multi-sensor data fusion model. The results show that the proposed PSOpid-LM-PSO provides a faster convergence and better fitness than other algorithms within the range-optimized search space with the system accuracy improved by approximately 18.6% compared to the classic LM algorithm. The contributions of this paper will reduce human effort in training the data fusion model using an on-line adaptation approach based on small changes from a prior trained model, or by using support vector machines for the one-to-many mapping approximation [27,28]. Another important area for further investigation is to explore the universal approximation capabilities of a standard multi-layer feed-forward neural network in most applications where numerous input samples need to be processed. Specifically, a two-hidden-layer feed-forward network using Kolmogorov’s theorem can be considered for approximating the high-dimensional data fusion model [29,30]. This well-optimized model for data fusing from the sensing information of homogeneous sensors is expected to support an enhancement in the synergistic accuracy.

## Figures and Tables

**Table 1:** Parameter information of the PSO method alone.

Parameters	Value
Swarm size (*Z_k_*)	28
Initial Position of Particles	Spread within a hypercube using a uniform random distribution
Minimum velocity norm	0.05
Inertial weight (*ω*)	1
Minimum position (min_pos)	−100
Maximum position (max_pos)	100

**Table 2:** Parameter information of the PSOpid-based method alone.

Parameters	Value
Minimum position (min_pos)	1.2 × min (LM-PSO)
Maximum position (max_pos)	1.2 × max (LM-PSO)
Proportional term (*K_P_*)	0.5 (fixed)
Integral term (*K_I_*)	0.5 (fixed)
Derivative term (*K_D_*)	0.6 (fixed)

**Table 3:** Comparison results of the algorithm enhancement rates (out of 100 independent runs).

Single-Subject Evaluation: Mean Distance Error [mm]					
Weight	Trilateration	LM alone	LM-PSO	PSOpid-LM-PSO	PSOpid-LM-PSO Error Reduction
58 kg	546.07	26.28	24.85	23.18	95.76%
64 kg	213.54	30.39	27.01	25.14	88.23%
72 kg	637.15	31.34	30.07	24.76	96.11%
85 kg	160.16	50.14	44.02	41.48	74.10%
90 kg	196.56	27.32	24.96	20.35	89.65%
